# The Function of Casual Sex Action and Inaction Regret: A Longitudinal Investigation

**DOI:** 10.1177/1474704921998333

**Published:** 2021-02-25

**Authors:** Leif Edward Ottesen Kennair, Trond Viggo Grøntvedt, Mons Bendixen

**Affiliations:** 1 Department of Psychology, 8018 Norwegian University of Science and Technology, Trondheim, Norway; 2 Department of Public Health and Nursing, HUNT Research Center, 8018 Norwegian University of Science and Technology, Levanger, Norway

**Keywords:** sexual strategies theory, inaction regret, action regret, casual sex, sociosexuality, sexual disgust, sex differences, adaptive function, longitudinal

## Abstract

In several recent papers the sex difference in regret predicted by sexual strategies theory has been supported: men more than women report regret passing up short-term sexual opportunities (inaction regret), while women regret having had sexual encounters (action regret). However, the adaptive function of regret, to improve future behavioral choices, has not been tested. In this first longitudinal test of behavioral change following regret, we consider whether regret actually results in adaptive shifts of behavior: will men who regret passing up sex engage in more short-term sex following regret? Will women who regret short-term encounters either choose better quality partners, reduce number of one-night stands or shift their strategy to long-term relationships? Across two waves (NT1 = 399, 65.4% women and NT2 = 222, 66.2% women) students responded to questions about casual sex action regret and inaction regret, along with possible outcomes, intrapersonal traits, and concurrent contextual predictors. There was no clear evidence for the proposed functional shifts in sexual behavior. Casual sex regret was associated with respondent sex and stable individual differences, such as sociosexual attitudes, regret processing and metacognitions, but the effect of these predictors were not consistent across the two waves. Among the tested concurrent contextual predictors, sexual disgust was the most consistent across waves. Regret is considered a gauge of the value and quality of the short-term sexual encounter. However, tentatively we conclude that after this first test of function using longitudinal data, we find no evidence of a mating strategy shifting effect following sexual regret.

## Introduction

Regret is a counterfactual cognitive-emotional process, where one reacts with aversive emotion while considering how much better it would have been if past behavioral choices had been different. Regret is generally presumed to have a positive function, being the most valued negative emotion (Saffrey et al., 2008): Regret is thought to aid in making sense of past events and providing insight into self, and also result in more adaptive future behavior. Experienced regret may thus be adaptive, if it actually improves future behavior (Zeelenberg, 1999).

Pieters and Zeelenberg’s (2007) theory of regret regulation emphasize that regret exist for behavioral regulation. While studies have linked regret to behavioral change, for instance in health behaviors (Brewer et al., 2016) and study habits (Valshtein & Seta, 2019), the majority of studies on regret investigate regret of hypothetical scenarios, anticipated regret in others (Connolly & Zeelenberg, 2002) or are retrospective (Seta & Seta, 2013). In this paper we will address *changes* in sexual behavior predicted by adaptive sexual regret for men and women. We will also test competing non-adaptive regret hypotheses, based on sociosexuality as a stable personality feature, and metacognitions about regret processing.

While most areas studied do not show sex differences in regret, the sexual domain does (Roese et al., 2006). Roese and colleagues (2006) found a moderate (d ≈ 0.40) sex difference in regret related to sexual inaction, with men regretting more than women having missed sexual opportunities. There were few other sex differences in regret toward friends and family, and none of these sex differences were comparable to that of sexual inaction regret. In line with this functional approach to regret, Galperin et al. (2013) suggested an adaptive function of short-term sexual regret: Regret about past sexual experiences may reduce future maladaptive sexual choices. This ‘feeling is for doing’ approach is also prominent in Roese and colleagues (2007) paper that points to regret serving to motivate improved future decisions. While several studies since then have considered Galperin et al.’s suggestions for proximate mechanisms (Bendixen et al., 2017; Kennair & Bendixen, 2018; Kennair et al., 2016, 2018), this specific adaptive function has yet to be tested empirically.

Sexual strategies theory (SST; Buss, 1998; Buss & Schmitt, 1993, 2017) is a theoretical approach providing explanations for sex differences based on parental investment theory (Trivers, 1972). The sexes are predicted to show behavioral differences especially in the sexual domain due to asymmetric minimal investment in offspring. For instance, the most investing sex is predicted to be choosier in specific preferences, such as partner’s willingness to invest in relationship, in partner acquisitions as the costs related to producing offspring is higher. In humans, women are the more investing sex in producing offspring with larger gametes, gestation, childbirth, and lactation. With regards to sexual encounters and regret, SST predicts sex differences in both action regret and inaction regret when the opportunity for having sex is present (Galperin et al., 2013). A brief and uncommitted sexual encounter could result in fitness increase for both women and men. However, due to the asymmetric costs, men have a substantially higher fitness gain from causal sex without investment compared to women. Sexual inaction has over evolutionary time been more fitness-reducing for men, and they are therefore expected to regret not engaging in non-committing sexual encounters more than women. As women carry the major costs of pregnancy that could result from casual sex without investment from partner, they are expected to experience more action regret.

The predictions derived from SST has been investigated in various studies since Roese and colleagues (2006). Galperin and colleagues (2013) found that women more than men reported action regret, men more than women reported inaction regret. No sex difference was found for other forms of regret (e.g., romantic nonsexual regret). This pattern has been replicated in other societies (e.g., Fisher et al., 2012), and more gender egalitarian samples (e.g., Kennair et al., 2016), and action regret in women seems to be specifically related to coital sex as opposed to other sexual behaviors (Eshbaugh & Gute, 2008). Within each sex, sociosexual orientation seems to influence the amount of regret, with less restricted individuals reporting less action regret (Kennair et al., 2016).

Unrestricted sociosexuality in women may increase opportunities to experience short-term sexual action regret, through having more one-night stands. Conversely, restricted men will more often pass up short-term sexual opportunities, thereby increasing their likelihood of experiencing inaction regret. Therefore, sexual personality may increase typical settings for sex typical regret. While such behavior is in accordance with their sociosexuality, the emotional consequences are not always in accordance with sociosexual orientation (Bendixen & Kennair, 2017; Kennair & Bendixen, 2012; Townsend & Wasserman, 2011). Further, to some degree prior behavior predicts future behavior, thus it may be that underlying sociosexuality and other traits maintain the short-term sexual behavior despite sex typical regrets and aversive emotional processing.

Galperin et al. (2013) did not specify what more adaptive future sexual behavior and choices would entail. Deciding what might be more adaptive sexual choices and behavior for women who regret having had one-night stands is not straightforward. For women regret seems to be driven by partner quality and sexual arousal, as women regret less when they take the initiative to having sex and regret more when experiencing disgust (Kennair et al., 2018). Thus, it is not necessarily merely a case of reducing number of one-night stands that might be the adaptive choice; similar behavior with better partners may reduce regret, too. Further, it might be that entering long-term committed relationship is the aim of the short-term behavior, and thus a predictable outcome for women with increased short-term sexual regret. What would be an adaptive behavioral shift for men after experiencing inaction regret is more obvious: If men have been selected to seize scarce and sought-after chances of reproductive opportunities, including short-term sex, this may explain why they experience inaction regret more than women after having had the chance of having a one-night stand or hook up. Consequently, men should seize their opportunities more often to increase the number of one-night stands.

Despite people’s intuition that regret is indeed helpful (Saffrey et al., 2008) regret might *not* facilitate better future behavioral choices. The question of whether regret is adaptive is closely related to whether other aversive emotional processing, such as rumination, is adaptive. While adaptationist theories of depression and rumination have been proposed (Andrews & Thomson, 2009; Bartoskova et al., 2018; Watson & Andrews, 2002), these have received critique from an evolutionary clinical perspective (Kennair et al., 2017; Nesse, 2018). A metacognitive approach (Wells, 2009) asserts that people have both negative metacognitions (rumination is uncontrollable and harmful) and positive metacognitions (rumination solves problems and prevents me from making future mistakes) (Papageorgiou & Wells, 2001a, 2001b). Changing these positive and negative metacognitions and discontinuing rumination is an efficient intervention against depressive disorder (Hagen et al., 2017).

If ruminative problem-solving was adaptive, such interventions should show no effect or adverse effects over time. Notably, the anti-depressive effect lasts across 1 and 3 years follow-up, with higher effect than competing therapy methods (Hjemdal et al., 2019; Solem et al., 2019). Solem et al. (2019) find that after discontinuing rumination there is improved work force, study participation, and improved quality of life at 3 years follow-up. Regret may be comparable to rumination, and not be adaptive despite adaptive hypotheses. It is possible that regret tracks evolutionary predictable contexts, that we can predict what men and women will regret in short-term sexual contexts (as predicted by SST), and still regret may not show any association with actual behavior changes. Rather, metacognitions or beliefs about regret might, as in depression, predict regret levels and regret processing, with no discernable adaptive shifts in behavior.

If regret results in less regrettable future behavior, the most obvious test is that current regret level and regret processing should predict less future regret. However, if one’s personality, in this case individual differences in sociosexuality, is a stable source for making choices that one later might regret, there will be less change of both behavior and resulting regret. Finally, if metacognitions about mental processing maintain regret, there will be no change in regret, even in the face of behavioral change—and this behavioral change may not be systematically directional.

## Aims and Hypotheses

We consider two different approaches to predict future adaptive change versus maintained regret, and we also predict of regret processing from a metacognitive non-adaptive processing perspective rather than an adaptive sexual behavior change perspective: The adaptive perspective to sexual regret suggests that sexual regret and regret processing might predict changes to sexual behavior in the future (Galperin et al., 2013). Galperin and colleagues did not specify what this might entail, however inaction regret should increase short-term sexual activity, especially in men. Action regret is harder to predict, as the adaptive shift in behavior might be to have less short-term sex, to enter a long-term relationship or to choose higher quality short-term partners, especially for women. Changes in sexual behavior in line with these predictions will support the adaptive sexual regret hypothesis, for each or both sexes. In general, present regret should result in less regrettable behavior and thus less future regret.

Opposed to this is the personality perspective where sociosexuality will account for stable behavior, despite sex typical regret: more restricted men will continue to have less short-term sex and continue to report sex typical inaction regret. Less restricted women will not show the above adaptive changes and continue to report action regret. Further, metacognitions will predict levels of regret processing, with no association with adaptive changes in sexual behavior. Support for these predictions will weaken the adaptive sexual regret hypothesis.

The following hypotheses are tested:

H1a: More inaction regret at T1 should increase more short-term sexual activity, especially in men.
H1b: More action regret at T1 should reduce short-term sex at T2, especially for women.
H2: More action regret at T1 should predict entering a long-term relationship at T2, especially for women.
H3: More action regret at T1 should predict higher short-term mate value partners at T2, especially for women.
H4a: In general, manifest functional regret must predict reduced future regret.
H4b: Nonfunctional regret will be maintained by intrapersonal stable traits such as sociosexual orientation, metacognitions, and regret processing independent of behavioral change.
H4c: Nonfunctional regret will be associated with previously established and new concurrent, contextual predictors of action regret including, disgust, gratification, intoxication, initiative, mate-value with no evidence of change at T1 and T2.

## Method

### Design and Participants

Students at a Norwegian university were invited to a panel study on uncommitted sex and responded to a web-based questionnaire in February-March 2019 (Time 1). Five-hundred twenty-nine students (63.2% women) gave their responses at Time 1 (T1). Following an e-mail invitation, *n* = 283 (53.5%) of these responded again in May-July 2019 (T2). The average time gap between the two measurement occasions were 4.5 months (134 days, between 101 and 187 days). The following criteria were used for inclusion: Reported age between 18 and 30 years (*n* = 20 dropped), student status ‘Yes’ (*n* = 23 dropped), self-reported sexual preference for opposite-sex partners (*n* = 32 dropped), and most recent intercourse with someone of the opposite sex (31 did not respond and 24 had sex with same-sex partner, *n* = 55 dropped). Eligible for analysis were *n* = 399 (261 women and 138 men) who responded at T1 and *n* = 222 (147 women and 75 men) who responded at T1 and T2 (55.6% of T1 respondents). The majority of these were not in a committed relationship at T1 (women: 59.6%, men: 71.5%, total sample: 63.7%).^1^

### Procedure

Research assistants enrolled at the bachelor program in psychology informed and invited students at various university faculties to the study at lectures and in campus hallways. Each student received a handout with information about the study, a link and a QR code to the online questionnaire and their rights as participants, including options for withdrawing from the study at any time after responding and before the data was made completely anonymous. The participants could activate the link and respond at their own leisure and encouraged to do this uninterrupted by others. When they activated the link, the following information about the study was stated: “We invite you to participate in a research project that examines students’ thoughts and feelings after having had casual sex (intercourse), and what factors that may affect these. Participating involved responding to an online questionnaire once or twice, now and again in approximately 4 months. Some of the questions are sensitive and relate to sexual acts and choices you may have made. Responding may cause some discomfort and embarrassment, and we recommend that all participants sit in an uninterrupted location when answering the questions. Each participant’s responses will not be recognizable in publications of the findings.” The procedure was approved by the Norwegian Center for Research Data (NSD), the National Competency Center for Data Protection in Research (Ref: 401757).

### Measurements

#### Regret

Participants were instructed to report their level of regret relating to casual sexual incidences and occasions they passed up having casual sex. Additional questions were posed regarding their most recent encounter, also how long ago this encounter took place (in months). These measures were based on Galperin et al. (2013) measure and has been applied repeatedly (Bendixen et al., 2017; Kennair et al., 2016, 2018). Participants read the following instruction: “Think about the times (or last time for most recent encounter) you had the experiences listed below. How do you feel about your actions/decisions?” The experiences for action and inaction regret respectively were: (1) “I had casual sex with someone,” and (2) “I passed up a chance to have casual sex with someone.” The following response options were given: I didn’t have the chance for casual sex (not coded), I had the chance, but did not have casual sex (not coded); I’m glad I did it (coded 0); Neutral—neither glad nor have regrets (1); I regret it somewhat (2); and I regret it very much (3).

#### Sociosexuality and short-term sexual activity

Participants completed a Norwegian translation of the revised Sociosexuality Orientation Inventory (SOI-R; Penke & Asendorpf, 2008). To better measure changes in number of recent sexual partners, the reference period for the first behavioral item measuring short-term sexual activity and new one-night stands was limited to 4 months (instead of the usual 12 months prevalence period). Internal consistency (Cronbach’s Alpha) for each of the three SOI-R components at T1 was good: Past SOI-Behavior (2 items, α = 0.89), SOI-Attitudes (3 items, α = 0.74), and SOI-Desire (3 items, α = 0.87). Corresponding internal consistency for behavior, attitudes, and desire at T2 was 0.85, 0.77, and 0.86, respectively. The scaling and scoring were equal to Penke and Asendorpf (2008). The sociosexuality components demonstrated high level of stability between T1 and T2 (*r* = 0.84, 0.69, and 0.69 for the behavior, attitudes, and desire components, respectively).

#### Partner quality (short-term and long-term attractiveness)

Along with ratings of their own global attractiveness as long-term and short-term partners (Bendixen, 2014), the participants rated the short-term and long-term attractiveness of their most recent one-night stand partner on a 7-item Likert scale with anchors –3 (*Well below average*) and +3 (*Well above average*) and midpoint 0 (*Average*). For this study, only their partner’s short-term attractiveness was applied.

#### Initiative

Participants rated their agreement on a 5-point Likert scale from 1 (*Strongly disagree*) to 5 (*Strongly agree*) to the following statement: ‘The last time they had casual sex I took the initiative to have sex.’

#### Disgust

Two items from Kennair et al. (2018) were used for measuring domain-specific sexual disgust. Participants rated their agreement to the following statements about the last time they had casual sex: ‘I felt the sex was disgusting’ and ‘I felt it was unhygienic’. Response alternatives were as for the Initiative measure. The two items correlated strongly (*r*_T1_ = 0.50 and *r*_T2_ = 0.60) and the item scores were averaged.

#### Gratification

Two items from the earlier studies by Kennair et al. (2016, 2018) were used for measuring physical gratification the last time they had casual sex. Participants rated their agreement on the above 5-point Likert scale on ‘general sexual pleasure’ and ‘achieved orgasm.’ The two items correlated strongly (*r*_T1_ = 0.54 and *r*_T2_ = 0.57). The item scores were averaged.

#### Intoxication

Similar to the above measures, participants rated their agreement to the following statement: ‘Last time I had casual sex I was drunk/intoxicated.’

#### Regret processing

This measure was constructed for the study and is based on the 22-item Ruminative Responses Scale (Treynor et al., 2003). The scale was adopted for use in the context of past casual sex encounters and covers both items of rumination about past choices and counterfactual thinking about more desirable behavior and covered 12 items. Participants rated how often that performed each statement (item) in a 5-point scale with response alternatives 1 (*Never*), 2 (*Almost never*), 3 (*Sometimes*), 4 (*Often*), and (*Almost all the time*). Internal consistency for the 12 items was excellent (α_T1_ = 0.93 and α_T2_ = 0.92). Items scores were averaged.

#### Metacognitions

Negative (NBRS) and Positive Beliefs (PBRS) about Rumination (Papageorgiou & Wells, 2001a, 2001b). We removed items that explicitly referred to depression, as that was not the aim of this study. This left us with four positive and 11 negative items. The participants reported their level of agreement on a 4-point response scale; 1 (*Do not agree*), 2 (*Agree slightly*), 3 (*Agree moderately*), and 4 (*Agree very much*). Internal consistency for the four PBRS items was good (α_T1_ = 0.76 and α_T2_ = 0.75). Internal consistency for the 11 NBRS items was good (α_T1_ = 0.82 and α_T2_ = 0.85). Items scores were averaged.

### Statistical Analyses

For predicting level of action and inaction regret, we applied Ordered Logistic Regression (OLR) analysis. The categorical regret variables have a natural ordering (low to high). However, when the distances between adjacent levels are unknown, as in this case, the OLR technique is preferable for ordinary least squares (OLS) regressions. OLR analyses produce ordered log-odds (logits) and proportional odds (OR). We report the latter along with 95% CIs and the test statistic *z*. Assumptions were checked for all analyses.^2^ Logistic regressions were applied for predicting dichotomous (No/Yes) outcomes, and OLS regressions for continuous outcomes. For analyses of change we regressed the predictors on the T2 outcomes scores, controlling for T1 (baseline) scores. Because of controversy and possible violation of basic assumptions (e.g., Allison, 1990; Cohen et al., 2003) we did not analyze change scores. All analyses were performed using Stata MP, version 16.1 (StataCorp., 2019) with robust estimations of standard errors.

### Data Availability

Data are available online (Bendixen et al., 2021).

## Results

### Dropout Analyses and Replication

Because we have panel data with two separate measurement occasions, we performed logistic regression analysis of dropouts (no/yes) to examine what factors affected likelihood of having dropped out from the study from T1 to T2 and if dropout was selective. Neither participant sex (*z* = 0.47), age (*z* = −0.40) or being in a committed relationship (*z* = 0.28) had any effect on likelihood of dropping out. Lifetime number of uncommitted sex partners (SOI-Behavior, 2 items) reduced the likelihood of dropping out (*z* = −2.01, *p* = .045), while neither recent number of sex partners during the past 4 months among those not in committed relationships (*z* = −0.39), SOI-Attitudes (*z* = −1.73, *p* = 0.73), nor SOI-Desire (*z* = −1.28, *p* = .201) predicted dropping out. Further, neither positive or negative beliefs about rumination (i.e., Metacognitions) significantly predicted dropout (*z* = 1.07, *p* = .283 and *z* = 1.79, *p* = .074, respectively). However, regret processing about the most recent casual sex encounter (*z* = 2.28, *p* = .022), general action regret (*z* = 2.25, *p* = .025), and most recent action regret (*z* = 2.53, *p* = .012) all significantly increased the likelihood of not responding at T2. To illustrate, while overall dropout rate was 44%, it was 38% among those who reported being glad for their most recent casual sex encounter, and 57% among those who strongly regretted most recent casual sex encounter. While dropout was selective, the overall level of casual sex regret at T1 was only 0.26 standard units (Cohen’s *d*) higher for the dropout group. In summary, dropout was more likely for participants with fewer lifetime uncommitted sex partners, for those who reported higher levels of regret processing, and who regretted casual sex more.

We ran ordered logistic regression analyses of action and inaction regret to test if prior findings on sex differences could be replicated in the current sample. For the most recent encounter, we found that men at T1 reported regretting having had sex significantly less than women (*n* = 380, *OR* = 0.57 [0.37–0.86], *z* = −2.67, *p* = .008), while men regretted passing up more than women (*n* = 344, *OR* = 2.12 [1.33–3.38], *z* = 3.15, *p* = .002).^3^ We re-ran the above analyses for most recent encounters at T1 on participants with complete data at both T1 and T2. Men regretted having had sex significantly less than women (*n* = 214, *OR* = 0.48 [0.26–0.86], *z* = −2.44, *p* = .015) and regretted passing up significantly more (*n* = 193, *OR* = 2.01 [1.06–3.83], *z* = 2.13, *p* = .033). Consequently, while dropout was not random, sex differences in action and inaction regret at T1 were not affected by dropout.

We tested Hypothesis 1a: “More inaction regret at T1 should increase more short-term sexual activity, especially in men” by looking at two types of short-term activity: (1) change in number of sex partners between T1 and T2, and (2) if the participants reported new sexual partners between T1 and T2 (no/yes). We included only those who reported *not* being in a committed relationship at T1. For the first analysis we applied an OLS regression predicting the number of recent sexual partners at T2 controlling for the effect of number of T1 recent sexual partners. Predictors were Sex, Age, Time between T1 and T2, and Inaction Regret. Men reported a stronger reduction in number of sex partners than women (*B* = −0.55, *t* = −2.00, *p* = .048). However, there was no effect of Inaction Regret (*t* = −0.35) nor any Inaction Regret × Sex interaction effect (*t* = 0.77). Age (*t* = −0.57) and Time (*t* = −1.06) had no effect on number of T2 recent partners. Correlation analyses showed that the number of recent casual sex partners was stable (*r* = 0.64). However, paired-sampled t-tests show that there was a significant overall reduction in level (number of partners for last 4 months) from T1 to T2 (*t* = 2.94, *p* = .004). This reduction was significant only for men (*t* = 3.24, *p* = .002) and moderately strong (*d* = 0.45). For women the reduction was negligible (*d* = 0.13). The bivariate association between most recent Inaction regret at T1 and number of recent partners at T2 was *r* = 0.22 for men (*r* = −0.10 for women), and in line with the prediction at first glance, however, since Inaction regret was similarly associated with number of recent partners at T1, the level of regret did not predict *change* in short-term sexual activity.

We applied logistic regression for predicting whether the respondents had new sexual partners or not between the two measurement occasions. Fully 68% of those not in a committed relationship reported at least one new partner between T1 and T2. There was no main effect of Sex (*z* = −0.68) or Inaction Regret (*z* = −0.30), or any Sex × Inaction Regret interaction effect (*z* = 0.88) on likelihood of having a new sexual partner. Accounting for the effect of number of T1 partners did not alter the above effects.

To test Hypothesis 1b: “More action regret at T1 should reduce short-term sex at T2, especially for women”, we re-ran the above OLS regression analysis substituting only Inaction Regret with Action Regret. When accounting for the effect of the other variables in the model, there was no effect of Sex (*t* = −0.44) or Action Regret (*t* = −0.67), and no Sex × Action Regret interaction effect (*t* = −1.15). Consequently, more action regret at T1 did not predict change in casual sex behavior. Neither Age (*t* = −0.74), nor Time (*t* = −1.15) had any effect on number of recent partners at T2. As shown in Table 1, the bivariate correlations between most recent Action Regret at T1 and number of recent partners at T2 was negative (*r* = −0.33). However, similar associations were evident for concurrent measures (T1), suggesting that the more one regrets the most recent encounter at T1 the less short-term sexual activity is reported at both T1 and T2.

**Table 1.** Zero-Order Associations (Pearson’s *r*) for Participants in Uncommitted Relationships and With Scores at T1 and T2.

Variable	1.	2.	3.	4.	5.	6.	7.
1. Time Lapse (T1–T2)	—						
2. T1 Recent Sex Partners	−0.09	—					
3. T2 Recent Sex Partners	−0.10	0.64	—				
4. T1 Action Regret	0.11	−0.29	−0.33	—			
5. T2 Action Regret	0.04	−0.10	−0.17	0.32	—		
6. T1 Inaction Regret	0.12	−0.01	0.02	−0.09	0.25	—	
7. T2 Inaction Regret	0.18	−0.02	−0.05	0.06	0.14	0.20	—

*Note*. *n* = 106.

We tested Hypothesis 2: “More action regret at T1 should predict entering a long-term relationship at T2, especially for women” by regressing Sex and Action Regret on likelihood of entering a committed relationship at T2 (i.e., having changed committed relationship status between T1 and T2 from ‘No’ to ‘Yes’). Of the 141 participants who reported not being in a committed relationship at T1, 29 had entered a committed relationship at T2. Significantly more women (26%) than men (12%) did so, χ^2^ (1, *N* = 141) = 4.11, *p* = .043. The logistic regression showed that the likelihood for entering a committed relationship was lower for men than for women (*OR* = 0.27, *z* = −2.47, *p* = .014), and that more Action Regret at T1 *reduced* the odds of having entered a committed relationship at T2 (*OR* = 0.51, *z* = −2.34, *p* = .019). The effect of Action Regret did not differ for men and women (no interaction).

We performed tests of Hypothesis 3: “More action regret at T1 should predict higher quality partners at T2 as measured by Short-Term Mate Value (STMV), especially for women” including participants who reported having had new casual sex partners between T1 and T2. First, correlation analysis showed no significant association between the T1 and T2 ratings of most recent partner short-term attractiveness (*n* = 105, *r* = 0.11, *p* = .28). A paired-samples t-test showed no overall change in STMV of partners between T1 and T2 (*t* = 0.42). Mean scores were 0.99 and 0.91 at T1 and T2, respectively, and reflect an overall positive evaluation of short-term attractiveness of partners. Next, we regressed Action Regret and Sex on perceived partner STMV at T2, controlling for perceived partner STMV at T1. The effect of Sex was marginal (*B* = −0.56, *SE* = 0.32, *t* = −1.74, *p* = .085) suggesting that relative to women, men found their new partner at T2 slightly less attractive relative to their T1 partner. However, most recent Action Regret was not associated with changes in perceived partner STMV (*t* = −1.04), and there was no significant Sex × Action Regret interaction effect (*t* = −0.61).

As an explorative test, we also analyzed perceptions of their partner’s long-term mate value (LTMV) at T1 and T2. First, there was no overall change in LTMV between T1 and T2 (*t* = −1.12). Level of attractiveness was average or below (Mean = −0.30 and −0.04 at T1 and T2, respectively). The T1 and T2 LTMV measures did not correlate (*r* = 0.12). The OLS regression suggests that men, relative to women, found their T2 partner’s less attractive than their T1 partner for a long-term relationship (*B* = −0.98, *SE* = 0.44, *t* = −2.23, *p* = .028). There was no effect of Action Regret (*t* = −1.30) nor any Sex × Action Regret interaction effect (*t* = −0.12). Consequently, regretting at T1 was not related to having sex with partner of higher mate value at T2 compared to T1 for those who had casual sex with a new partner.

We tested Hypothesis 4a: “In general, manifest functional regret must predict reduced future regret” in several ways. First, we cross-tabulated the T1 and T2 scores for Action Regret for those who reported new sexual partners. Level of Action Regret at T1 was clearly related to level of Action Regret at T2, χ^2^(9, *N* = 103) = 19.76, *p* = .019. The association was small-to-moderate (*r*_tau_ = 0.22, *r*_polychoric_ = 0.37) and stronger for women (*r*_tau_ = 0.37, *r*_polychoric_ = 0.58) than for men (*r*_tau_ = 0.10, *r*_polychoric_ = 0.15). Paired-samples t-tests showed that there was no overall change in Action Regret from T1 to T2 (*t* = 0.87). However, women’s scores were significantly *reduced* between T1 and T2, *t*(64) = 3.01, *p* = .004, *d* = 0.37, while men’s scores *increased* marginally, *t*(37) = −1.97, *p* = .057, *d* = 0.32. Still, those who regretted their most recent sexual encounter at T1 (score 2 and 3), tended to regret less at T2 while the opposite pattern was evident for those who were ‘glad’ or ‘neither glad or regretted’ (scores 0 and 1). This pattern of change and relative level of stability can reflect regression toward the mean. Statistically, this is the more parsimonious interpretation of these changes.

Second, we regressed Sex, Relationship Status at T1 (not committed vs committed), and Action Regret at T1 on T2 Action Regret (*n* = 102) using Ordered Logistic Regression. There were main effects of Sex (*OR* = 8.77, *z* = 3.04, *p* = .002) and T1 Action Regret (*OR* = 2.63, *z* = 2.71, *p* = .007), and Relationship Status (*OR* = 6.44, *z* = 2.22, *p* = .027). Men and those in a committed relationship regretted more at T2 relative to T1. When accounting for the above factors, there was no Sex × T1 Action Regret interaction effect (*z* = −1.57, *p* = 0.117).

Finally, for Inaction Regret, the T1 and the T2 scores were associated, χ^2^(6, *N* = 174) = 24.40, *p* < .001. The stability of Inaction Regret was small-to-moderate (*r*_tau_ = 0.20, *r*_polychoric_ = 0.35), and similar women and men. There was no overall change from T1 to T2 Inaction Regret (*t* = 1.23). For Inaction Regret, almost half of those who were ‘glad’ they passed up at T1 shifted toward neither glad nor regretted or regretted somewhat (score 1 and 2) at T2. For the remaining groups, there was a shift toward less regret passing up at T2 relative to T1. When predicting T2 Inaction regret there was no effect of Sex or Relationship Status on Inaction Regret at T2, also not Inaction Regret at T1 (*z* = 1.66, *p* = .097). Hence, stability in Inaction Regret was low across encounters of passing up casual sex.

When we tested Hypothesis 4b: “Nonfunctional regret will be maintained by intrapersonal stable traits such as sociosexual orientation, metacognitions, and regret processing independent of behavioral change” we omitted any measures of behavior change because the first hypothesis was unsupported. The bivariate association between Sociosexual Attitudes, Action Regret, Regret Processing, and Metacognitions at T1 and T2 (Table 2) guided the inclusion of predictors in each model. All analyses were performed on participants who reported having had a new casual sex partner at T2. For T1 Action regret (n = 103) we included Sex and concurrent measures of Sociosexual Attitudes and Regret Processing. Having more restricted sociosexual attitudes (*z* = −3.35, *p* = .001) was the primary predictor of Action Regret in this model, with marginal effects of Sex (*z* = −1.86, *p* = .062) and Regret processing (*z* = 1.92, *p* = .055). For T2 Action Regret (*n* = 105) we included Sex and the concurrent measures of positive metacognitions and regret processing. Positive metacognitions predicted more Action Regret (*z* = 2.35, *p* = .019), and more regret processing predicted more Action Regret (*z* = 2.09, *p* = .037). There was no effect of Sex (*p* = .113).

**Table 2.** Zero-Order Associations (Pearson’s *r*) for Participants Reporting on New Sex Partner Between T1 and T2.

Variable	1.	2.	3.	4.	5.	6.	7.	8.	9.	10.
1. T1 SOI-Attitudes	—									
2. T2 SOI-Attitudes	0.68	—								
3. T1 Positive Metacognitions	−0.06	−0.02	—							
4. T2 Positive Metacognitions	−0.08	−0.00	0.66	—						
5. T1 Negative Metacognitions	−0.22	0.02	0.27	0.28	—					
6. T2 Negative Metacognitions	−0.13	−0.07	0.29	0.39	0.53	—				
7. T1 Regret Processing	−0.22	−0.10	0.33	0.31	0.22	0.22	—			
8. T2 Regret Processing	−0.19	−0.32	0.19	0.26	0.04	0.10	0.35	—		
9. T1 Action Regret	−0.35	−0.29	0.10	0.14	0.17	0.08	0.30	0.12	—	
10. T2 Action Regret	−0.10	−0.12	0.20	0.31	0.05	−0.07	0.18	0.32	0.27	—

*Note*. *n* = 103; SOI = Sociosexual Inventory.

For testing Hypothesis 4c: “Nonfunctional regret will be associated with previously established and new concurrent, contextual predictors of action regret including, disgust, gratification, intoxication, initiative, mate-value with no evidence of change at T1 and T2”, we followed the same analytic strategy as for Hypothesis 4b. Guided by the bivariate associations (Table 3) we included in addition to Sex, concurrent measures of disgust, gratification, initiative, and partner’s STMV as predictors of Action Regret at T1 (*n* = 102), and disgust, gratification, and partner’s STMV as predictors of Actions Regret at T2 (*n* = 105). For Action Regret at T1, all predictors except Sex had a significant effect on Action Regret. At T2, disgust and lack of gratification both predicted Action Regret. As evident from Table 4, disgust was the most consistent predictor for regretting having had casual sex. The effect of lack of gratification was stronger at T1 than at T2, and partner’s short-term mate value was significant only at T1.

**Table 3.** Zero-Order Associations (Pearson’s *r*) for Participants Reporting on New Sex Partner Between T1 and T2.

Variable	1.	2.	3.	4.	5.	6.	7.	8.	9.	10.	11.	12.
1. T1 Sexual Disgust	—											
2. T2 Sexual Disgust	0.43	—										
3. T1 Sexual Gratification	−0.36	−0.10	—									
4. T2 Sexual Gratification	−0.15	−0.33	0.43	—								
5. T1 Intoxicated	0.13	−0.04	−0.10	−0.00	—							
6. T2 Intoxicated	0.04	0.14	0.02	−0.11	0.27	—						
7. T1 Sexual Initiative	−0.09	−0.08	0.09	0.13	−0.17	−0.03	—					
8. T2 Sexual Initiative	−0.04	−0.15	0.10	0.11	−0.16	−0.05	0.37	—				
9. T1 Partner’s STMV	−0.49	−0.28	0.25	0.09	0.02	−0.09	−0.12	0.06	—			
10. T2 Partner’s STMV	−0.16	−0.52	0.11	0.37	0.11	−0.06	0.16	0.27	0.13	—		
11. T1 Action Regret	0.49	0.23	−0.46	−0.23	0.01	−0.06	−0.20	−0.06	−0.42	−0.15	—	
12. T2 Action Regret	0.21	0.53	−0.11	−0.32	0.05	0.12	−0.08	−0.13	−0.23	−0.39	0.28	—

*Note*. *n* = 101; STMV = Short-Term Mate Value.

**Table 4.** Ordered Logistic Regression for Most Recent Casual Sex Regret Encounter at Time 1 and Time 2) for Participants Reporting on New Sex Partner.

	Time 1			Time 2		
	*OR (SE)*	*Z*	*p*	*OR (SE)*	*Z*	*p*
Sex	0.37 (0.25)	−1.49	.137	2.09 (1.26)	1.23	.219
Sexual Disgust	2.02 (0.65)	2.17	.030	2.77 (1.17)	2.41	.016
Sexual Gratification	0.57 (0.14)	−2.38	.017	0.59 (0.16)	−1.90	.057
Partner’s STMV	0.54 (0.10)	−3.46	.001	0.75 (0.14)	−1.51	.132
Sexual Initiative	0.61 (0.15)	−2.06	.039	(omitted)		

*Note*. *n* = 101. STMV = Short-Term Mate Value

## Discussion

Many emotions have evolved, including fear (Cannon, 1915; Kennair, 2007; Marks & Nesse, 1994) and disgust (Al-Shawaf et al., 2018; Tybur et al., 2009). Galperin et al. (2013) suggested an adaptive function of men’s sexual inaction regret and women’s sexual action regret, where both sexes should make more adaptive future sexual behavior choices, based upon the aversive emotional component of their regret. In general, action and inaction regret should result in more adaptive behavior, less regrettable behavior and thus less regret. This is the first empirical test of this functional hypothesis of regret.

We found no support for the hypothesis that inaction regret should increase short-term sexual activity, as inaction regret at T1 did not increase number of one-night stands or the likelihood of having a new one-night stand for either men or women between T1 and T2. We neither found support for the hypothesis that action regret at T1 would result in fewer short-term sexual partners at T2, especially for women. In addition, and contrary to the functional hypothesis, level of casual sex regret at T1 *reduced* the odds of entering a committed relationship at T2. Furthermore, the level of regret about having had casual sex was not associated with changes in perceived partner short-term attractiveness between T1 and T2 for those reporting a new casual sex partner (Hypothesis 3). This was also true for perceived partner long-term attractiveness (post-hoc, explorative analysis). Finally, there was no clear-cut conclusion based on the analysis that regret is a manifest functional mechanism that results in reduced future regret. Women reported lower levels of action regret at T2 relative to T1, while men reported more. Still, those who regretted more at T1 reported lower levels of regret at T2, but this may also be an effect of regression toward the mean as those who reported being ‘glad’ they had casual sex (or passed up having casual sex) reported more regret at T2.

The competing hypotheses that regret is non-functional and maintained by stable, intrapersonal factors or a result of concurrent, contextual factors were supported. The first of these suggests that sociosexuality, metacognitions and regret processing, independent of behavioral change, would predict regret. However, these predictors were not consistent across waves. Action regret showed moderate association with sociosexual attitudes at T1, but weak at T2. Regret processing was moderately associated with action regret across both waves, while positive and negative metacognitions were less consistently associated. The newly developed measure of regret processing is probably closely related to the personality trait neuroticism. As such, it is possible that these more stable traits and individual differences explain who will regret having had sex: more neurotic individuals will process negative aspects more and less restricted individuals will experience less reason to regret a physical encounter.

Of the concurrent, contextual factors included in the study (sexual disgust, sexual gratification, intoxication when having had sex, sexual initiative, and partner’s short-term mate value), only sexual disgust consistently predicted action regret across both waves, while sexual gratification was stronger associated with less regret at T1 relative to T2. We also found that higher partner short-term mate value and taking the initiative reduced regret at T1. Again, this replicates the gist of previous research into proximate mechanisms (Kennair et al., 2016, 2018). However, without any identified behavioral change above, we are left with the conclusion that in addition to some effect of personality, what we find is that regret largely is a dynamic gauge of whether the casual sex being evaluated was good or bad.

An important aspect of a functional emotion is that it should produce change in behavior, and thus reduce the necessity of experiencing the emotion. Pain due to a stone in one’s shoe should motivate removing said stone, and thereby discontinuing the pain. Fear of a venomous spider should motivate avoidance and reduce the present level of fear. Without behavioral change as a result of an internal state there is no interaction with reality, and thus nothing for selection to work on. It was therefore surprising, from a functional perspective, that regret as counterfactual cognitive-emotional process was both continuous and relatively stable across different one-night stands for the same participants. There was further little evidence of behavioral change, which of course may be because we have not managed to define or operationalize this well enough. However, in sum, the tentative conclusion after the current investigation is that regret is to some degree maintained by individual differences and a result of concurrent, contextual factors, rather than a process that changes behavior in any predictable, functional direction.

It seems that men do not change their mating strategy after regretting having passed up casual sex opportunities some months earlier. Future research is therefore needed to further investigate men’s strategy shifts as a function of regret. However, while the preliminary conclusion needs to be tentative, there is no evidence in current data to suggest a function of inaction regret. For women there is more evidence in the literature that a maintained short-term strategy (more short-term sex) is associated with increased emotional discomfort (Townsend et al., 1995; Townsend & Wasserman, 2011). Thus, the conclusion that negative emotions do not necessarily motivate a change in sexual strategy might be considered more robust. However, that begs the question of why regret exists, given that it shows evidence of sex specific responses, as predicted by sexual strategies theory (Buss, 1998; Buss & Schmitt, 1993, 2017). One possible explanation is that different mental adaptations have different and uncoordinated effects on different behaviors: Tendencies toward regret and engaging in short-term mating are influenced different individual differences in both neuroticism and emotional lability as well as sociosexuality and other mental mechanisms that motivate sexual behavior within different domains (Kennair et al., 2015; Meston & Buss, 2007, 2009). All of these will not increase personal happiness (Buss, 2000). Another explanation may be that modern mating scene is evolutionary mismatched (Goetz et al., 2019). Finally, many mental and emotional responses exist despite not being functional. For example, rumination probably does not solve problems (Kennair et al., 2017), while discontinuing rumination seems to actually increase adaptive behavior as measured by increased quality of life and improved workforce participation or study activity 3 years after treatment (Solem et al., 2019). Panic disorder exists, but in different countries how patients misinterpret, in a positive feedback loop, bodily sensations of anxiety such that in some Arabic countries they will perceive a Djinn sitting on their chest while in Western countries people may fear a heart attack. Since neither perception is correct, despite systematic symptoms, panic disorder is primarily merely a misinterpretation disorder (Clark, 1986; Kennair, 2007). While worry might be a good anti-confirmation bias program at low levels, the worry involved in Generalized Anxiety Disorder is debilitating, not functional, and discontinuing worry provides efficient treatment (Kennair et al., 2020; Nordahl et al., 2018). Any functional explanation of the disorder will therefore be incorrect, although many of the underlying mental mechanisms involved may be adaptations (Nesse, 2018). However, we need to consider that despite the current findings, there may be other explanations and functional aspects of short-term sexual regret that may be discovered through more thorough and formal analysis of the design feature and behavioral outcome of a functional regret program. We suggest that two recent theoretical papers—Lukaszewski et al. (2020) and Al-Shawaf et al. (2016)—might aid this conceptual functional analysis.

One of the most surprising findings is that action regret reduces the likelihood of entering a long-term relationship. This was in the opposite direction of our functional prediction. It is possible that some of the more successful one-night-stands resulted in long-term relationships over time, or that regret was increased when one at some level desired a long-term relationship from the short-term encounter, but this did not happen, although it is not possible to discern these processes from the available data. Another possibility is that underlying personality factors cause those who are happier with their short-term experiences to be more positive toward other romantic relationships. Neuroticism decreases long-term relationship satisfaction (Gerlach et al., 2018), and might conceptually, given our current finding of the effect of regret processing, also, be associated with dissatisfaction after short-term encounters.

Recent studies of proximate predictors of the sex difference in action and inaction casual sex regret have suggested that a high degree of disgust is associated with higher levels of regret (Kennair et al., 2018). Within each sex there is an effect of sexual gratification (Kennair et al., 2016), and particularly among women who take the initiative to have casual sex (Kennair et al., 2018). In the current findings these factors were not as robust, however, for some analyses we had few participants. Despite this, given the current findings, bad sex will increase regret, good sex or a sexy partner will decrease regret—which thus may act as an online emotional and cognitive gauge of one’s experience. Evolved sexual psychology, as predicted by SST, will influence that process and evaluation based on sex specific likelihoods and thresholds for what is considered desirable or what is adaptive. However, much as our ability to track our relationship satisfaction in long-term relationships dynamically and online (Conroy-Beam et al., 2015, 2016), also based upon our evolved sexual psychology, we might track discrete sexual encounters more with emotional-cognitive processing akin to regret or rumination. We might use terms like satisfaction or dissatisfaction about long-term or ongoing processes, and regret about discrete choices and events, such as short-term sexual encounters. However, as in our relationships, we do not necessarily always make decisions based directly on this gauge of satisfaction, and other personality features, including or level of satisfaction may decide whether we stay or leave a long-term relationship or change our behavior within the relationship. Actually, reasons why we think we stay or leave and what we actually do are probably not as closely connected as people believe (Machia & Ogolsky, 2020).

### Limitations and Future Research

While this is the first longitudinal investigation of behavioral changes following regret, there are some limitations that need to be addressed. First, the time lapse between the first and the second measurement was only 4.5 months on average. Although two-third of the single participants reported at least one new one-night stand during this period, this may be too short a time, or involve too few encounters for any adaptive mechanism to be activated. Despite the longitudinal design of the study, the reports on the most recent sexual encounters are retrospective in nature, and therefore subject to possible response biases. Second, there seems to be some self-selection and selective dropout at follow up. Relative to those reporting at T1 only, those with complete data at both T1 and T2 reported having had more casual sex, and less regret and regret processing at T1. Still, this selective dropout did not affect the relative sex differences in action and inaction regret at T1. However, the overall lower level of regret for those with complete data most likely have constrained the variance in regret in the longitudinal analyses increasing the risk of false negatives. This is sustained by the relatively low number of cases eligible for analysis. Finally, one important limitation is that we did not measure ambivalent feelings for action regret or passing up opportunities for having sex. By following the Galperin et al. (2013) measurement approach, we forced people to either be happy with their decision, neutral (neither happy or regret), regret somewhat or regret strongly. Most people may be more ambivalent, though, and may therefore describe their regret best along two dimensions: (1) degree of satisfaction with their choice, and (2) degree of regret/dissatisfaction for making the same choice. Future studies on sexual regret may want to include measures capturing this ambivalence to examine changes in either or in both these aspects of choice to have a one-night stand or to pass up.

As the first investigation of the function of inaction and action regret, hypothesized by Galperin and colleagues, we need to be cautious: as we note above, there may be other functions or other operationalizations of Galperin et al.’s ideas. These need to be considered both in depth theoretically and in future empirical investigations.

Finally, we have presented a new measure of regret processing, which needs to be tested further in future studies. The scale measures ones’ processing of negative past choices and counterfactual processing of more desirable behaviors. The scale was highly internally consistent and moderately stable across waves with different partners. This scale may provide a better measure of regret after discrete experiences. We expect that the scale to large degree correlates with trait neuroticism and recommend also measuring this trait specifically in future research.

## Conclusions

After this first test of the function of action and inaction short-term sexual regret, we must conclude that there is no evidence of a predicted, functional effect on sexual behavior. Rather intrapersonal measures seem to some degree predict regret of the latest short-term sexual encounter. Further, the level of regret is largely stable over time, despite new partners, but best explained by concurrent, contextual measures. Despite the current findings, Galperin et al. (2013) sparked several papers that have mapped proximate factors and that may explain sex differences in short-term sexual regret (Bendixen et al., 2017; Kennair et al., 2016, 2018).

Perhaps future research will better operationalize the function of action and inaction casual sex regret. However, tentatively we conclude that we find no evidence of a mating strategy shifting effect. People do regret a lot of actions and inactions in their life, however, very few people exercise more, eat healthier, spend less, or act more sensibly due to regretting such behavior; indeed, these behaviors are only problematic if they are maintained—which alas often is exactly the case. While many emotional-cognitive processes are adaptive, probably all are not.

## Notes

1. The participants opted for one of seven statuses: Single, single with occasional one-night stands, friends with benefits, and open sexual relationship without commitment (all coded as 0 = Single/uncommitted). Married, going steady, or in a committed and exclusive sexual relationship (all coded as 1 = Committed).

2.
https://stats.idre.ucla.edu/stata/dae/ordered-logistic-regression/

3. For ‘the times’ they had experienced casual sex, men again reported regretting less than women (OR = 0.40 [0.26–0.61], z = −4.30, p < .001), but regretting passing up more (OR = 3.41 [2.08–5.60], z = ¬4.85, p < .001). Time elapsed since their most recent one-night stand (Mean = 12 months, range 0–94 months) was not significantly associated with level of regret.
